# Usefulness of Endoscopic Managements in Patients with Ceftriaxone-Induced Pseudolithiasis Causing Biliary Obstruction

**DOI:** 10.1155/2017/3835825

**Published:** 2017-11-02

**Authors:** Yasuhiro Doi, Yasushi Takii, Hiroyuki Ito, Norihiko Jingu, Kentaro To, Sinichiro Kimura, Koichi Kimura, Kensaku Sanefuji, Hirofumi Ikeda, Sayaka Tachibana, Takeshi Otsuka

**Affiliations:** ^1^Department of Internal Medicine, Munakata Medical Association Hospital, Fukuoka, Japan; ^2^Department of Surgery, Munakata Medical Association Hospital, Fukuoka, Japan; ^3^Department of Nephrology, Munakata Medical Association Hospital, Fukuoka, Japan

## Abstract

Ceftriaxone (CTRX) is known to cause reversible biliary stones/sludge, which is called biliary pseudolithiasis. We report two rare cases of biliary obstruction by pseudolithiasis shortly after completing CTRX treatment. Stones and sludge, which had not been detected before CTRX administration, appeared in the gallbladder and common bile duct and led to biliary obstruction and acute cholangitis. The obstructions were successfully treated with endoscopic retrograde biliary drainage and endoscopic sphincterotomy. CTRX-induced biliary pseudolithiasis has been reported mainly in children and adolescents but is also seen in adults with similar incidence rate. Although CTRX-induced biliary pseudolithiasis is usually asymptomatic and disappears spontaneously after discontinuing the drug, some patients develop biliary obstruction. Endoscopic managements should be considered in such cases.

## 1. Introduction

Ceftriaxone (CTRX), a widely used third-generation cephalosporin, has a broad spectrum and a long half-life of 6 to 9 hours [1]. In some cases, CTRX is known to precipitate with calcium ion in the biliary tract. The precipitation usually disappears after discontinuation of CTRX and is thus called biliary pseudolithiasis. Since the first report in 1986 [2], biliary pseudolithiasis caused by CTRX has been reported mainly in children and adolescents but also in adults [3, 4]. Although the calcified lesions usually disappear spontaneously after discontinuation of CTRX, they sometimes cause acute cholangitis, acute cholecystitis, or acute pancreatitis, requiring interventions.

We report two cases of biliary obstruction by CTRX-induced biliary pseudolithiasis that required endoscopic managements.

## 2. Case Presentation

### 2.1. Case 1

**Figure 1:**
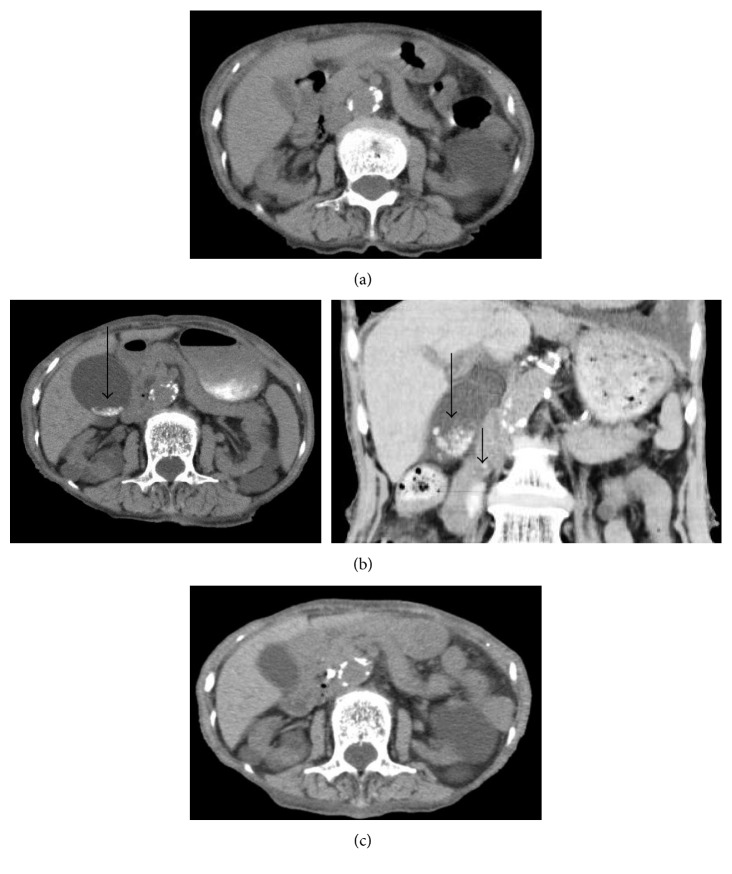
CT scans in Case 1. (a) Small stones or sludge were not detected in the biliary tract before administration of CTRX. (b) (*Left*, axial view; *right*, coronal view) small stones and sludge appeared in the gallbladder (long arrows) and common bile duct (short arrow) 3 days after ending CTRX administration. (c) The small stones and sludge disappeared after 4 weeks.

**Figure 2:**
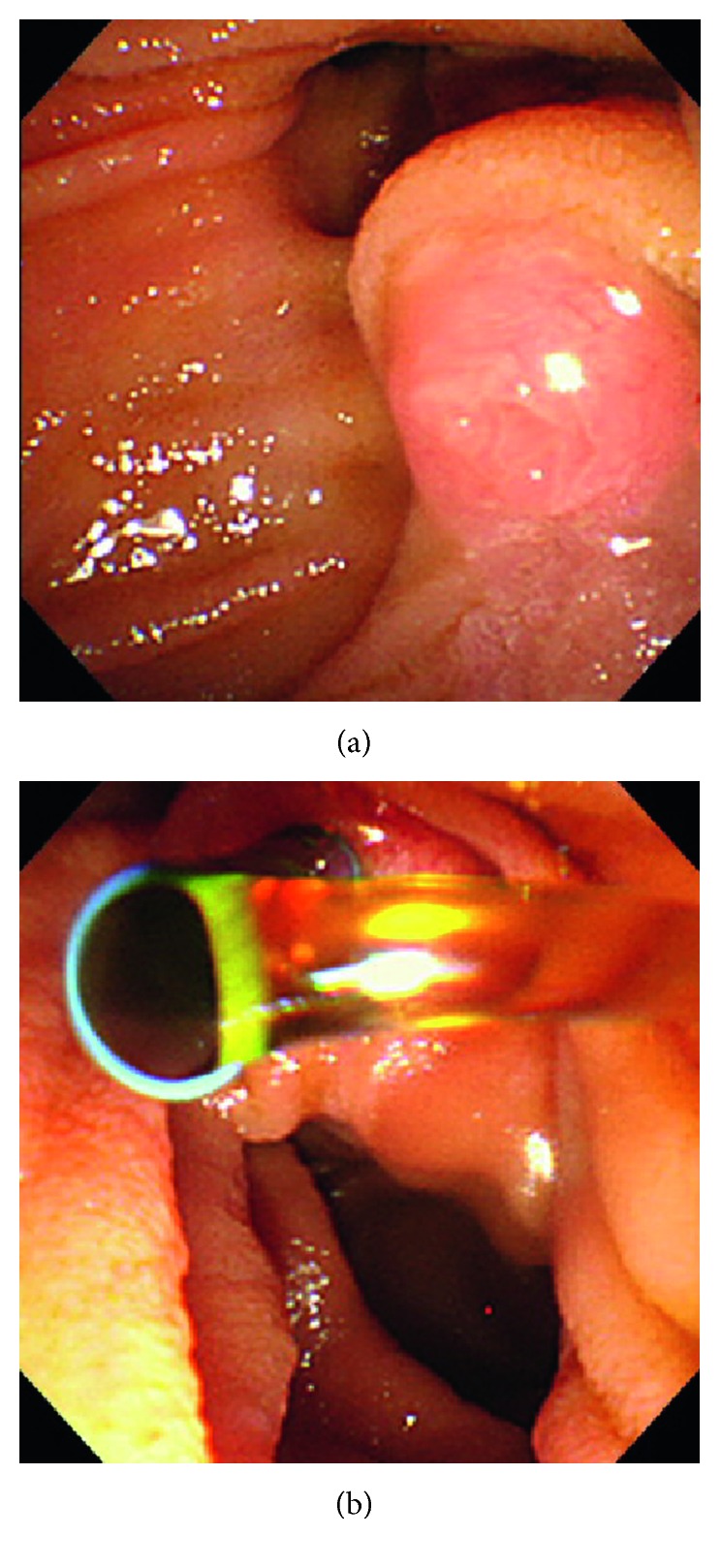
Endoscopic views before (a) and after (b) ERBD in Case 1. (a) Absence of bile excretion from the ampulla of Vater indicated bile congestion by common bile duct obstruction. (b) Insertion of biliary stent resulted in excretion of the bile.

A 91-year-old woman with stage 5 chronic kidney disease possibly due to hypertensive nephrosclerosis or chronic glomerulonephritis was admitted to the Department of Nephrology of our hospital because of a 2-week history of dyspnea. Physical examination on admission was not remarkable except for oxygen saturation of 91% on 2 L/min of oxygen and pretibial pitting edema. Laboratory examinations showed white blood cell (WBC) count of 5,300/μL (reference range (RR): 3,300–8,600), C-reactive protein (CRP) 2.17 mg/dL (RR: 0–0.14), blood urea nitrogen 80.3 mg/dL (RR: 8.0–20.0), serum creatinine 5.69 mg/dL (RR: 0.46–0.79), aspartate aminotransferase (AST) 24 U/L (RR: 13–30), alanine aminotransferase (ALT) 9 U/L (RR: 7–23), lactate dehydrogenase (LDH) 276 mg/dL (RR: 124–222), alkaline phosphatase (ALP) 326 U/L (RR: 103–322), γ-glutamyl transpeptidase (γ-GTP) 42 U/L (RR: 9–32), total bilirubin 0.6 mg/dL (RR: 0.4–1.5), and serum amylase 104 U/L (RR: 44–132). Chest X-ray and computed tomography (CT) scan revealed ground-glass appearance of the bilateral lung field and consolidations of the right middle lobe and left lower lobe. The patient was diagnosed with pulmonary edema and pneumonia and was treated with 2 g of CTRX intravenously every 24 hours for 10 days along with intravenous furosemide. Three days after ending the administration of CTRX, she complained of right upper abdominal pain. Laboratory data demonstrated worsening of the liver function: AST 232 U/L, ALT 46 U/L, LDH 481 mg/dL, ALP 866 U/L, γ-GTP 167 U/L, total bilirubin 0.6 mg/dL, and serum amylase 115 U/L. CT scan revealed small stones and sludge in the gallbladder and common bile duct, which had not been detected in the CT scan on admission (Figures 1 and 1). A diagnosis of grade II cholangitis due to CTRX-induced biliary pseudolithiasis was made, and the patient was referred to the Department of Internal Medicine. Endoscopic retrograde cholangiography (ERC) demonstrated the absence of bile excretion from the ampulla of Vater, indicating bile congestion by obstruction of the common bile duct (Figure 2). Endoscopic retrograde biliary drainage (ERBD) by inserting a biliary stent resulted in excretion of the bile (Figure 2), and cholangitis resolved after the procedure. CT scan after 4 weeks revealed disappearance of the small stones and sludge in the gallbladder and common bile duct (Figure 1), and the biliary stent was withdrawn the following day.

### 2.2. Case 2

**Figure 3:**
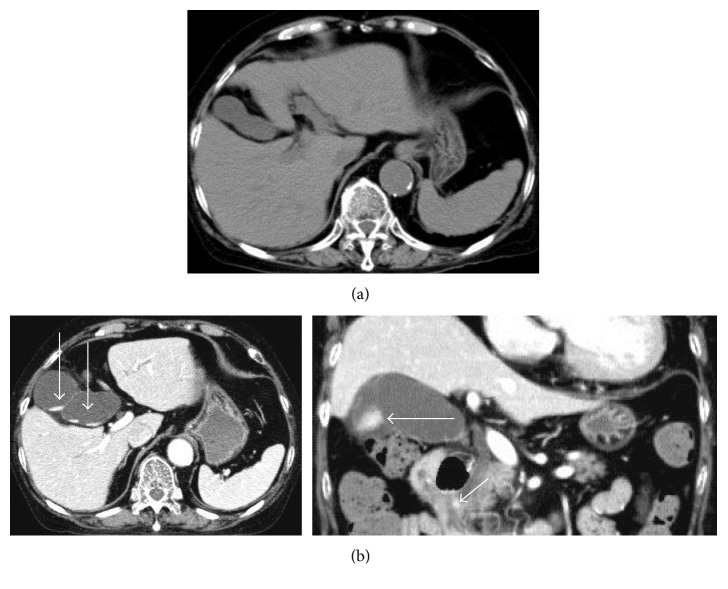
CT scans in Case 2. (a) Small stones or sludge were not detected in the biliary tract before administration of CTRX. (b) (*Left*, axial view; *right*, coronal view) small stones and sludge appeared in the gallbladder (long arrows) and common bile duct (short arrow) 10 days after ending CTRX administration.

**Figure 4:**
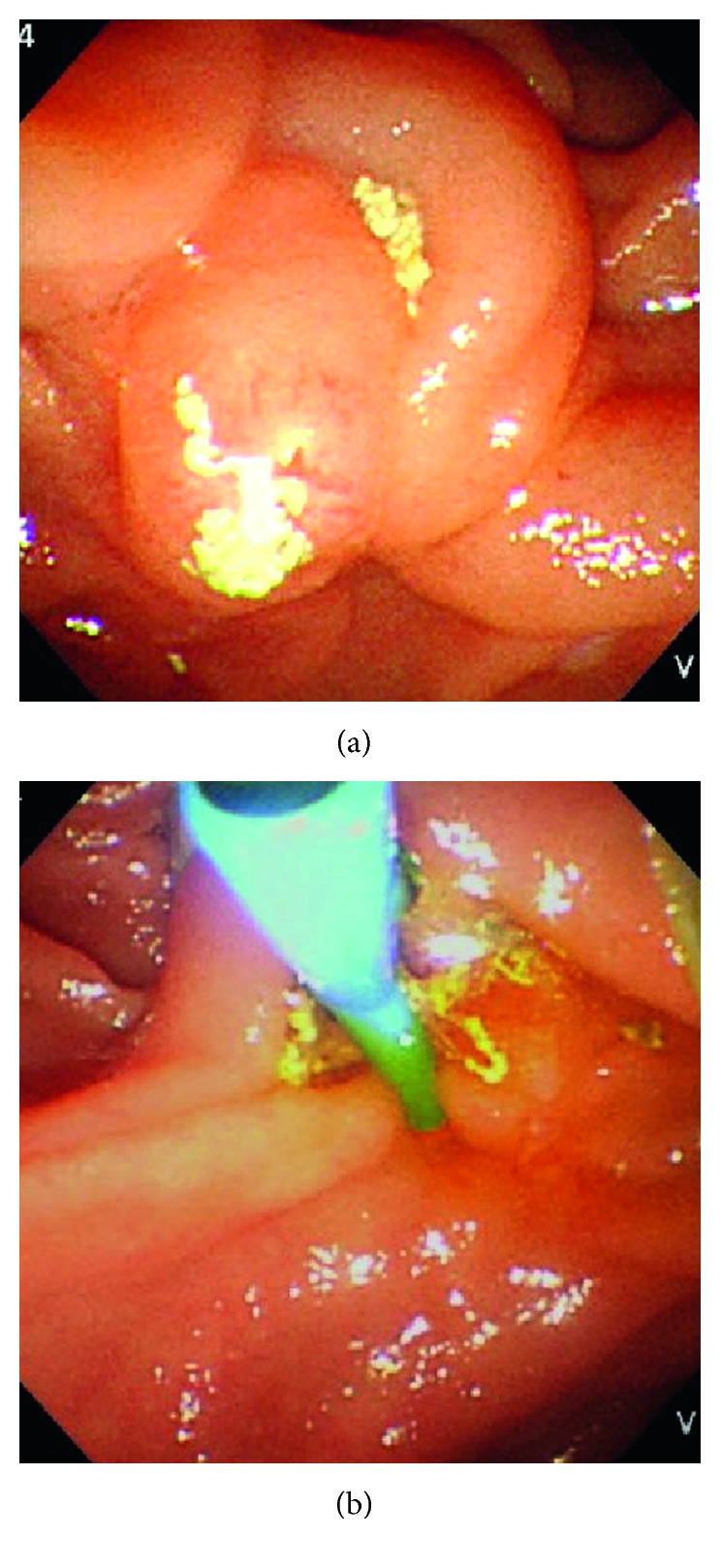
Endoscopic views before (a) and after (b) EST and ERBD in Case 2. (a) Deposition of white sludge and absence of bile excretion were seen on the ampulla of Vater. (b) EST and ERBD led to excretion of the bile.

An 82-year-old woman visited the hospital because of diarrhea and mild abdominal pain in the umbilical region. She had a medical history of dermatomyositis, interstitial pneumonitis, diabetes mellitus, and chronic heart failure with diastolic dysfunction and a relatively low left ventricular ejection fraction of 50% and was receiving 9 mg of prednisolone and 2 mg of tacrolimus. A diagnosis of acute enteritis was made. In addition to fasting and intravenous hydration, she was treated with 2 g of intravenous CTRX every 24 hours for 5 days, and subsequently with oral levofloxacin at 500 mg once daily for 5 days after campylobacter had been cultured from the stool obtained on admission. She recovered fully and was discharged on day 13. Two days after discharge, she complained of severe upper abdominal pain and back pain. Laboratory data showed leukocytosis (WBC count 14,000/μL), elevation of CRP (1.42 mg/dL), mild liver dysfunction (AST 47 U/L, ALT 18 U/L, LDH 235 U/L, ALP 233 U/L, γ-GTP 73 U/L, total bilirubin 0.5 mg/dL), and elevations of serum amylase (938 U/L) and lipase (4113 U/L, RR: 12–52). CT scan revealed small stones and sludge in the gallbladder and common bile duct, which had not been detected two weeks earlier (Figure 3). She was diagnosed with grade II acute cholangitis and grade 1 acute pancreatitis. Emergent ERC showed deposition of white sludge on the ampulla of Vater and absence of bile excretion (Figure 4). Endoscopic sphincterotomy (EST) and ERBD were performed, resulting in excretion of the bile (Figure 4) and resolution of the abdominal pain. Hydration required for the treatment of acute pancreatitis exacerbated the heart failure, making the treatment of acute pancreatitis difficult. The patient developed multiple organ failure and died on day 17.

## 3. Discussion

We experienced two cases of common bile duct stones and sludge causing acute cholangitis shortly after completing CTRX treatment, which required endoscopic managements. Acute pancreatitis was also seen in Case 2. In both cases, biliary stones or sludge had not been detected in the CT scan 2 weeks before the onset. In Case 1, the stones and sludge spontaneously disappeared 4 weeks later. The white fine precipitation observed in Case 2 was different from the appearance of ordinary biliary stones consisting mainly of cholesterol or bilirubin. These observations strongly indicated that CTRX induced the biliary stones and sludge. Previous studies demonstrated that intravenously administered CTRX is excreted to the bile as a divalent anion and can precipitate with calcium ion to form calcium-CTRX complex at a dose of 2 g or higher [1, 5–8].

Cases of pseudolithiasis have been reported mainly in children and adolescents with an incidence of 17–57% after CTRX administration [9–14]. In most cases, biliary pseudolithiasis was detected between 3 and 14 days after initiating CTRX administration and completely resolved within a few weeks after discontinuing CTRX. Biliary symptoms, such as right upper abdominal pain, nausea, and vomiting, were reported in 0–20% of these cases. Although the CTRX-induced biliary pseudolithiasis is less well studied in adults, the clinical features including the incidence rate are similar to those for children and adolescents [4, 15, 16].

Interventional procedures are required in some cases of CTRX-induced biliary pseudolithiasis when the stones or sludge induce biliary obstruction, leading to acute cholangitis or acute pancreatitis as in our cases [7, 17–20]. Endoscopic procedures, such as EST and/or ERBD, are the treatment of choice in such cases because they are less invasive than surgical procedures. Most of the previous reports performed surgery [7, 17–19], and there is only one case report in the English literature which reported endoscopic management for CTRX-induced biliary pseudolithiasis [20]. Because discontinuation of CTRX usually leads to spontaneous resolution of biliary pseudolithiasis, we believe that cholecystectomy should be considered only when biliary obstruction persists even after discontinuing the drug and endoscopic managements are difficult. This point is supported by a case of an 18-month-old boy with CTRX-induced cholecystitis, where stones in the gallbladder detected during CTRX administration were not found in the gallbladder resected 9 days after discontinuing the drug [21].

## 4. Conclusion

CTRX-induced biliary pseudolithiasis is usually asymptomatic and disappears spontaneously after discontinuation of the drug, making the complication easily overlooked especially in adults. However, some patients develop biliary obstruction requiring emergent procedures, and thus caution is necessary during CTRX administration. Once biliary obstruction occurs, endoscopic managements such as EST and ERBD should be considered.
